# Immunomodulatory Effects of *Bifidobacterium* spp. and Use of *Bifidobacterium breve* and *Bifidobacterium longum* on Acute Diarrhea in Children

**DOI:** 10.4014/jmb.2206.06023

**Published:** 2022-08-11

**Authors:** Yae Jin Choi, Seon-Hee Shin, Hea Soon Shin

**Affiliations:** 1 College of Pharmacy, Duksung Women’s University, Seoul 01369, Republic of Korea; 2 Department of Pediatrics, Hallym University College of Medicine, Hwaseong 18450, Republic of Korea

**Keywords:** Cytokine regulatory effect, *Bifidobacterium* spp., acute diarrhea

## Abstract

The intake of probiotic lactic acid bacteria not only promotes digestion through the microbiome regulated host intestinal metabolism but also improves diseases such as irritable bowel syndrome and inflammatory bowel disease, and suppresses pathogenic harmful bacteria. This investigation aimed to evaluate the immunomodulatory effects in intestinal epithelial cells and to study the clinical efficacy of the selected the *Bifidobacterium breve* and *Bifidobacterium longum* groups. The physiological and biochemical properties were characterized, and immunomodulatory activity was measured against pathogenic bacteria. In order to find out the mechanism of inflammatory action of the eight viable and sonicated *Bifidobacterium* spp., we tried to confirm the changes in the pro-inflammatory cytokines (TNF-α, interleukin (IL)-6, IL-12) and anti-inflammatory cytokine (IL-10), and chemokines, (monocyte chemoattractant protein-1, IL-8) and inflammatory enzymatic mediator (nitric oxide) against *Enterococcus faecalis* ATCC 29212 infection in Caco-2 cells and RAW 264.7 cells. The clinical efficacy of the selected *B. breve* and *B. longum* group was studied as a probiotic adjuvant for acute diarrhea in children by oral administration. The results showed significant immunomodulatory effects on the expression levels of TNF-α, IL-6, IL-12, MCP-1, IL-8 and NO, in sonicated *Bifidobacterium* extracts and viable bifidobacteria. Moreover, each of the *Bifidobacterium* strains was found to react more specifically to different cytokines. However, treatment with sonicated *Bifidobacterium* extracts showed a more significant effect compared to treatment with the viable bacteria. We suggest that probiotics functions should be subdivided according to individual characteristics, and that personalized probiotics should be designed to address individual applications.

## Introduction

During infection with pathogenic microorganisms, the human body secretes pro-inflammatory cytokines, and as a result, immune cells remove these microorganisms by inducing inflammatory reactions [1]. In addition, the production of nitric oxide (NO) and prostaglandin E2 (PGE2) in response to infections causes various inflammatory reactions such as pain, swelling, and fever. [2]. However, persistent inflammatory reactions may cause damage to mucous membranes, leading to many dysfunctions [3]. Macrophages produce anti-inflammatory cytokine, interleukin-10 (IL-10), which promotes humoral immune responses, and suppresses the immune responses by inhibiting pro-inflammatory cytokines like tumor necrosis factor-alpha (TNF-α) [4]. Furthermore, probiotics that increase the ability of macrophages to recognize pathogens are known to promote the secretion of immune substances with immunomodulatory effects in the intestine.

Probiotics were defined by the World Health Organization as “living microorganisms that improve host health when administered at an effective level” such as lactic acid bacteria (LAB) including *Lactobacillus*, *Bifidobacterium*, and *Streptococcus* species [5]. Currently, four types of LAB are recognized, probiotics, prebiotics, synbiotics, and postbiotics, depending on the ingredients. Among them, postbiotics defined by The International Scientific Association of Probiotics and Prebiotics (ISAPP) as “inanimate microorganisms that confer a health benefit on the host which include some non-living microorganisms, whether it be whole microbial cells or cell components” [6]. Many researchers have reported various physiologic activities of probiotics, such as immune activity enhancement by LABs, inhibitions of the proliferation of intestinal putrefactive bacteria and pathogens, anticancer, anti-inflammatory, and antiviral effects, and reductions in blood cholesterol levels [7, 8]. In addition, LABs reinforce the ability of macrophages to recognize and kill harmful bacteria in the gut, promote or suppress the secretion of cytokines in intestinal tract to regulate immune reactions, and participate in inflammatory reactions to regulate the immune system [9, 10].

The bacteria belonging to the genera, *Lactobacillus* and *Bifidobacterium* are considered to be significant in the context of intestinal microbiome because they are resistant to the gastrointestinal environment and readily form colonies by attaching to the intestinal tract. In addition, they produce various antimicrobial peptides such as bacteriocin, lactic acids, acetic acids, and other metabolites of them while living in intestinal microflora. Although most studies have been conducted with viable LABs, it has been revealed that inactivated (non-viable) cells of that LABs are better adhered to the digestive system, have health benefits, and contain antibacterial active substances such as large quantities of peptidoglycan (a cell wall glycoprotein), lipopolysaccharides (LPS), and organic acids [11]. Also, probiotic stability can be ensured during storage, and the inactivated cells required can be supplied in quantity more readily than viable cells [12, 13]. Inactivated LABs contain various bioactive metabolites, and are viewed as postbiotics in the intestinal microbiome. On the other hand, bifidobacteria is a Gram-positive anaerobic bacterium, that predominates during the neonatal period and reduces with age. Probiotic bifidobacteria can create healthy intestinal acidic environments, which may prevent or reduce the severities of intestinal tract infections [14]. Combined administration of antibiotics and probiotic bifidobacteria as a potential immune-adjuvant has been recommended for patients that develop chronic intestinal microbiome imbalance due to long-term antibiotic administration [15].

Probiotics are often administered as alternative treatments for infectious diarrhea and the maintenance of intestinal homeostasis [16]. Also, postbiotics collected from healthy individuals have been administered to treat diarrhea in young children and alleviate symptoms of various diseases such as atopic dermatitis in adults [17]. Infectious diarrhea is caused by pathogenic bacteria or viruses, and it is classified as acute or chronic according to whether the condition lasts less or greater than two weeks. Acute diarrhea rapidly causes symptoms such as vomiting, fever, and abdominal pain, and when excessive, may lead to electrolyte imbalance and life-threatening dehydration [18]. Fernandez *et al*. also demonstrated the anti-rotaviral effects of *Bifidobacterium adolescentis* and *Lactobacillus casei*, which could be used as antiviral probiotics [19]. Probiotic bifidobacteria has been used as an alternative to relieve infectious diarrhea and improve intestinal homeostasis [20].

To develop probiotics and postbiotics that possess these functions, the present study was conducted to evaluate against *Enterococcus faecalis* ATCC 29212 infection and whether sonicated bifidobacterial metabolites or viable cells are effective. In this investigation, we investigated the immune modulatory effects of eight species of viable or sonicated *Bifidobacterium* strains by examining their effects on pro-inflammatory cytokines such as TNF-α, interleukin-6 (IL-6), and IL-12, and anti-inflammatory cytokines, such as IL-10, and chemotactic cytokines, referred to as chemokines, like Monocytes chemoattractant protein-1 (MCP-1), and IL-8 and inflammatory enzymatic mediators, like nitric oxide (NO). In addition, we investigated whether sonicated bifidobacterial metabolites influence NO production and compared the clinical characteristics and efficacies of *B. breve* and *B. longum* in children with acute diarrhea.

## Materials and Methods

### Cell Lines and Culture

The human colon adenocarcinoma cell line Caco-2 and the murine macrophage cell line RAW 264.7 were obtained from the American Type Culture Collection and Korea Cell Line Bank. Caco-2 cells were grown in Roswell Park Memorial Institute 1640 medium (RPMI 1640, BD Difco, USA) containing 10% fetal bovine serum (FBS; Gibco, USA) with 1% penicillin and streptomycin (10,000 U/ml, Gibco) at 37°C in a humidified 5% CO_2_ incubator. Cultured pathogenic *E. faecalis* ATCC 29212 was grown in Brain Heart Infusion (BHI) broth medium (BD Difco) at 37°C to achieve a concentration of 108~109 the colony forming unit (CFU)/ml. RAW 264.7 cells were grown in Dulbeccós Modified Eaglés Medium (DMEM, USA) containing 10% FBS with 1% penicillin and streptomycin at 37°C in humidified atmosphere with 5% CO_2_ incubator.

### Probiotic Bacterial Strains

Eight species of *Bifidobacterium* spp. were obtained from the Korean Collection for Type Cultures (Table 1). These bacteria were cultured at 37°C for 48 h in Lactobacilli MRS Broth (Man, Rogosa & Sharpe, Difco.) with 0.05% (w/v) of L-cysteine under anaerobic chamber (Baker Ruskinn, UK, CO_2_ 5%, H_2_ 5%, N_2_ 90%) at 37°C for 48 h. The pathogenic Gram-positive bacteria, *E. faecalis* ATCC 29212 was tested and stored at -70°C in nutrient broth (BD Difco) with 20% glycerol before testing. Cultured pathogenic bacteria were grown in Brain Heart Infusion (BHI) broth medium (BD Difco) at 37°C to achieve a concentration of 1 × 10^8^ the colony forming unit (CFU)/ml. To prepare sonicated-inactivated *Bifidobacterium*, cells were harvested during the exponential growth phase by centrifugation at 4,000 ×*g* for 10 min, washed with phosphate buffered saline (PBS, Gibco Invitrogen), and suspended in the same buffer. The bacterial suspensions were adjusted to a final concentration of 1 × 10^9^ CFU/ml, and sonicated under for six cycles using a sonicator (VCX 130, Sonics & Materials, Inc.) under the following conditions (amplitude, 100%; pulse on, 60s; and pulse off, 60s). Sonicated bifidobacteria were centrifuged at 8,000 ×*g* for 10 min, and filtered with a 0.22 μm cellulose acetate filter (Millipore, USA) to use for experiment. Viable bifidobacterial supernatants were resuspended in PBS at a concentration of 1 × 10^9^ CFU/ml without sonicating the cells.

### Cytotoxicity Assay

The cytotoxicity of the sonicated *Bifidobacterium* extract on cultured cells was assessed using MTT [3-(4,5-dimethylthiazol-2yl)-2,5-diphenyltetrazolium bromide] assay (Sigma-Aldrich, USA). RAW 264.7 cell was seeded on 96-well plates at a density of 1 × 10^4^ cells/well with 10% (v/v) eight species of viable or sonicated *Bifidobacterium* extracts, and analyzed after 24 h for both cells. The cells without bifidobacteria were used as the control for 100% of viability. The medium was removed, and 100 μl of MTT solution was added to each well. After incubation for 3 h at 37°C, the MTT solution was aspirated, and 100 μl of dimethyl sulfoxide (DMSO) was added to each well. Viable cells were detected by measuring absorbance at 540 nm.

### Cytokine Expression by *Bifidobacterium* spp.

To evaluate the secretion of cytokines, after pathogenic *E. faecalis* (1 × 10^9^ CFU/ml) was spread on the Caco-2 cells of solid agar medium in a 37°C for 24 h, the sonicated *Bifidobacterium* extract and viable bifidobacteria were added with LPS (1 μg/ml). We measured six types of cytokines through this assay which are immune mediators, the pro-inflammatory cytokines (TNF-α, IL-6, and IL-12), and anti-inflammatory cytokine (IL-10), and chemotactic cytokines, referred to as chemokines (MCP-1, and IL-8). The measurements were performed via human enzyme-linked immunosorbent assay (ELISA) kits (BD Bio Science, USA). The Caco-2 cells were incubated in 96-well plates until the confluence reached 80% or more. A total of 100 μl/well LPS (1 μg/ml, Sigma-Aldrich) was added to the cultured cells, and they were then incubated at 37°C for 30 min. After incubation, 10%(v/v) sonicated *Bifidobacterium* extracts, and viable bifidobacteria were added to each well, and incubated for 1 h; LPS (1 μg/ml) was then added to each well. The incubated supernatant (100 μl) was added to a microplate coated with the primary antibody on the previous day, incubated at room temperature for 2 h, and washed five times with a wash buffer. After washing, the plates were treated with 100 μl of secondary antibody solution for 1 h. After incubation with the secondary antibodies, the substrate solution was added, and the plate was incubated in the dark for 30 min. The culture supernatants contained in tetra-methylbenzidine were taken 100 μl and reacted in the dark room, and then a stop solution was added 50 μl to terminate the reaction. The cells in the negative control group were incubated with medium alone, and those in the positive control group was treated with LPS alone. The absorbance was measured at 450 nm using a microplate reader (Molecular Devices, USA). The calibration curves were created using serially diluted solutions containing human six types of cytokines such as TNF-α, IL-6, IL-12, IL-10, MCP-1, and IL-8 used to quantify cytokine release in the supernatants.

### Nitric Oxide (NO) Assay

The inflammatory enzymatic effects of the sonicated *Bifidobacterium* extracts were confirmed using an NO assay. RAW 264.7 cells were seeded on 96-well plates at a density of 1 × 10^5^ cells/well, and pathogenic *E. faecalis* (1 × 10^9^ CFU/ml) was spread at 37°C for 24 h, After incubation, the culture medium was removed, and the cells were washed twice with PBS. The eight species of sonicated *Bifidobacterium* extracts were treated to the cells at 10% (v/v) concentration. The cells were incubated at 37°C for 48 h with LPS (1 μg/ml). Then, the supernatant of each well (100 μl) was transferred into a new 96-well plate with equivalent quantity of Griess reagent (Stock-I: 0.2%naphthalene diamine HCl, stock-II: 2% sulfanilamide in 5% H_2_PO_4_; Sigma-Aldrich) were used as materials. The new plate was incubated for 10 min at room temperature, and evaluated with a microplate reader at 540 nm.

### Clinical Efficacy of *B. breve* and *B. longum* Treatment

Human studies conducted in this study was approved by the Institutional Review Board at Duksung Women's University (IRB approval number; 2021-012-017-B). Among children aged 3 months to less than 5 years admitted to a university hospital from November 2021 to February 2022, in stool specimens from 50 pediatric patients with acute diarrhea. After obtaining informed consent for participation, these patients were included in the study to investigate the effect of *B. breve* and *B. longum* on acute diarrhea in children. A 10-fold dilution of each fecal sample was produced by adding approximately 100 mg of phosphate-buffered saline (PBS, pH 7.4, Sigma); the sample was centrifuged at 3,000 rpm for 20 min at 4°C, and the supernatant was taken and used. The participants were randomly divided into three groups. The *B. breve* group (*n* = 17) and *B. longum* group (*n* = 19) were administered 0.5 × 10^8^ CFU/ml of *B. breve* and *B. longum* orally twice a day, and non-fat dry milk powder was administered in the placebo group (*n* = 14). The *B. breve* ATCC 42255, *B. longum* ATCC 11207, and placebo were manufactured by Cell Biotech R&D Center. Pediatric patients who had acute diarrhea with a duration of seven days or less, and who had diarrhea once or more within 24 h before admission were included in the evaluation. The efficacy was evaluated by examining the number of diarrhea episodes, fever, and vomiting at the same time periods every day after admission. The duration of diarrhea from admission to the last diarrhea were recorded.

### Statistical Analysis

Values a re expressed as the mean ± standard deviation (SD). For statistical evaluation of data, one-way or two-way, ANOVA tests were applied, with SPSS statistical program (Windows version 25.0, IBM, USA). Differences were assessed significant at *p* < 0.05 for all comparisons by Tukey test.

## Results

Studies on the immunomodulatory effects of probiotics in the gastrointestinal tract indicate their effects are inadequate because most probiotics do not reach the gut alive and viable LABs are likely to be inactivated by antibiotics [21]. For these reasons, we evaluated sonicated bifidobacterial metabolites and viable cells. Inactivated LAB could increase probiotics by additionally supplying bioactive compounds to the LAB already present in the intestinal microbiome.

### Cell Viability

**Fig. 1:**
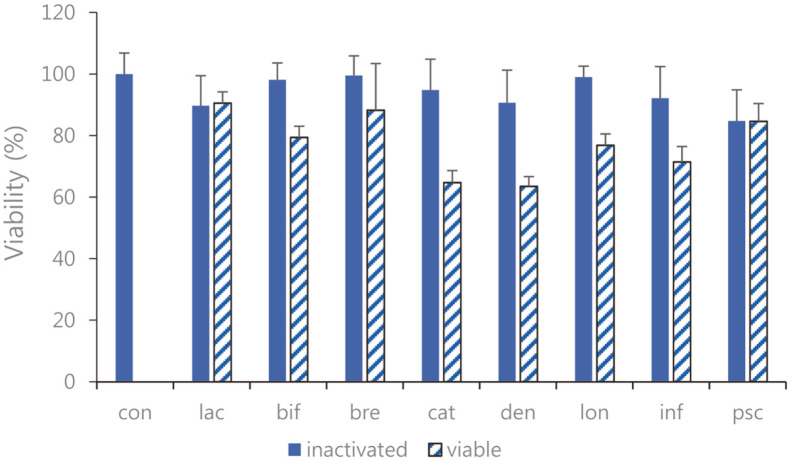
Viability of cultured RAW 264.7 cells with the eight species of sonicated *Bifidobacterium* extracts and viable bifidobacteria. The cells were treated with 10% (v/v) sonicated *Bifidobacterium* extracts for 24 h. Cell viability was performed by an MTT assay. con: control; lac: *B. lactis*; bif: *B. bifidum*; bre: *B. breve*; cat: *B. catenulatum*; den: *B. dentium*; lon: *B. longum*; inf: *B. infantis*; psc: *B. pseudocatenulatum*. Values are represented as the mean ± S.D.

Performing the MTT assay, we investigated the cell cytotoxicity of *Bifidobacterium* strains on RAW 264.7 cells. Cell viability after treatment with sonicated *Bifidobacterium* extracts showed approximately 95% viability compared to that observed in the control group. The cell viability for viable bifidobacterial supernatants was lower than for the inactivated (sonicated) bifidobacterial extracts, which is estimated to have affected cell survival conditions due to the pH environment lowered to lactic acid, organic acid, peptidoglycans, lipoteichoic acids, and teichoic acids produced by bifidobacterial metabolism (Fig. 1).

### Measurement for Quantification of Cytokines

The ingestion of probiotics has many benefits, but probiotics are particularly effective for managing inflammatory intestinal diseases. IL-6, IL-12, and TNF-α (pro-inflammatory cytokines) and the chemokines IL-8 and MCP-1 are known to regulate immunity by inducing inflammatory reactions [22]. However, when overexpressed, they can induce excessive inflammatory reactions and cause autoimmune diseases or even cancer. Macrophages produce the anti-inflammatory cytokine IL-10, which suppresses immune responses by inhibiting TNF-α [23]. A number of studies have shown that ingestion of probiotics inhibits inflammation by modulating the expression of inflammatory cytokines in the intestines. Probiotics are largely composed of anaerobic bacteria and cannot survive under aerobic conditions, which considerably disadvantages the use of viable forms for drug formulations. As a result, recent research efforts have tended to focus on the immunomodulatory effects of postbiotics. Furthermore, the use of non-viable microbial cell extracts to treat wounds has shown that inactivated postbiotic bifidobacteria are more effective than viable forms in some subjects [24].

**Fig. 2:**
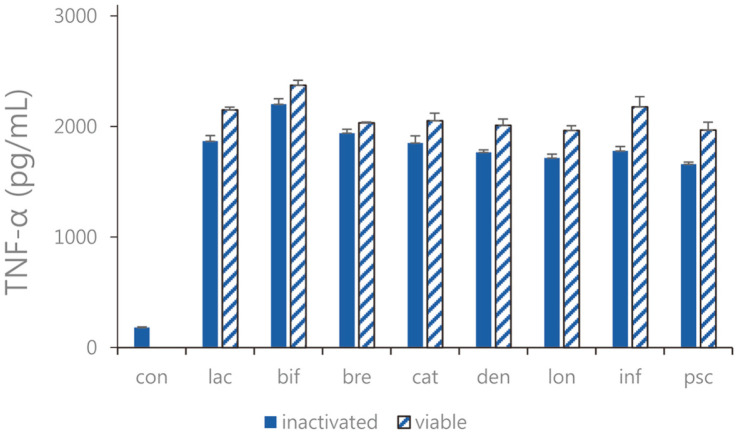
TNF-α expression of the sonicated *Bifidobacterium* and viable bifidobacteria in Caco-2 cells treated with pathogenic *E. faecalis* ATCC 29212. con: control; lac: *B. lactis*; bif: *B. bifidum*; bre: *B. breve*; cat: *B. catenulatum*; den: *B. dentium*; lon: *B. longum*; inf: *B. infantis*; psc: *B. pseudocatenulatum*. Values are represented as the mean ± S.D.

**Fig. 3:**
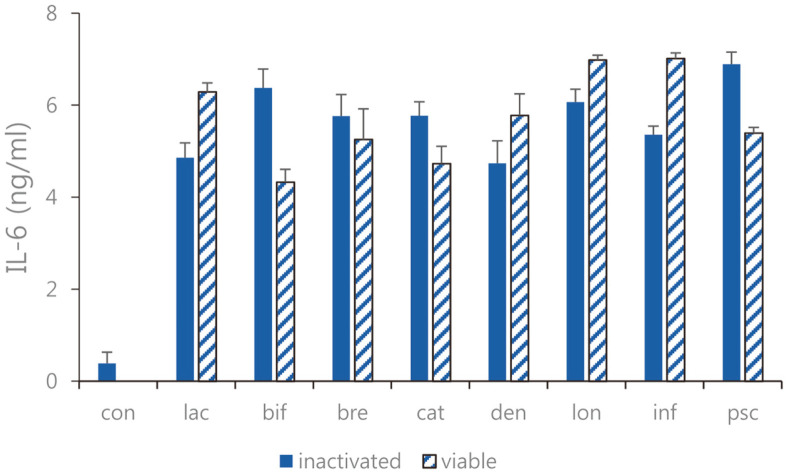
IL-6 expression of the sonicated *Bifidobacterium* and viable bifidobacteria in Caco-2 cells treated with pathogenic *E. faecalis* ATCC 29212. con: control; lac: *B. lactis*; bif: *B. bifidum*; bre: *B. breve*; cat: *B. catenulatum*; den: *B. dentium*; lon: *B. longum*; inf: *B. infantis*; psc: *B. pseudocatenulatum*. Values are represented as the mean ± S.D.

**Fig. 4:**
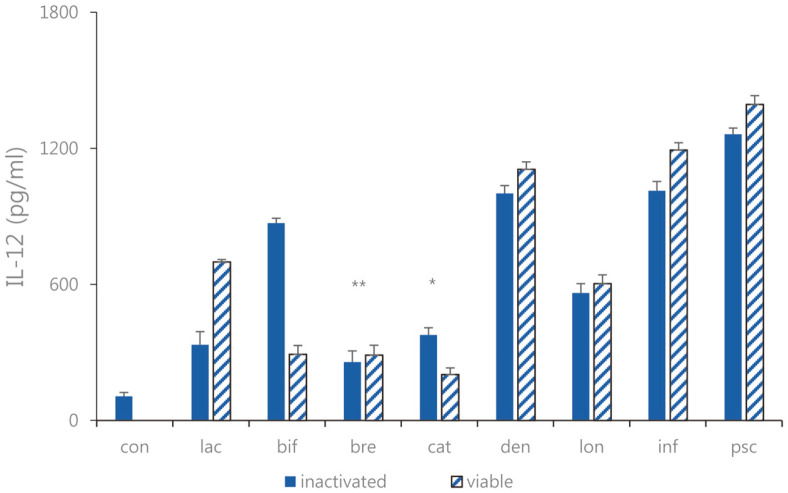
IL-12 expression of the sonicated *Bifidobacterium* and viable bifidobacteria in Caco-2 cells treated with pathogenic *E. faecalis* ATCC 29212. con: control; lac: *B. lactis*; bif: *B. bifidum*; bre: *B. breve*; cat: *B. catenulatum*; den: *B. dentium*; lon: *B. longum*; inf: *B. infantis*; psc: *B. pseudocatenulatum*. Values are represented as the mean ± S.D. (*p < 0.05, **p < 0.01).

Expression of TNF-α, IL-6, and IL-12 as pro-inflammatory cytokines were measured. In Caco-2 cells, the expression levels of TNF-α, IL-6, and IL-12 were decreased (Figs. 2-4). Overall, the TNF-α inhibitory effects of sonicated *Bifidobacterium* extract were not superior to but similar to those of all eight species of viable bifidobacteria. Moreover, the expression levels of IL-6 in cells treated with sonicated *Bifidobacterium* extracts and viable bifidobacteria were both decreased compared with those in the control group. Among the four lactic acid bacteria, *B. lactis*, *B. bifidum*, *B. breve*, and *B. longum*, and *B. longum*, the difference in IL-6 expression was the most obvious when viable bifidobacteria and sonicated bifidobacterium extract were inoculated. In Caco-2 cells treated with sonicated *Bifidobacterium* extracts and viable bifidobacteria, IL-12 expression decreased, and *B. breve* and *B. catenulatum* showed particularly significant inhibition by 257.4. ± 49.32 pg/ml and 365.62 ± 47.61 pg/ml. Among the four lactic acid bacteria, *B. lactis*, *B. bifidum*, *B. breve*, and *B. catenulatum*, and *B. breve*, the difference in IL-12 expression was the most obvious when viable bifidobacteria and sonicated bifidobacterium extract were inoculated.

**Fig. 5:**
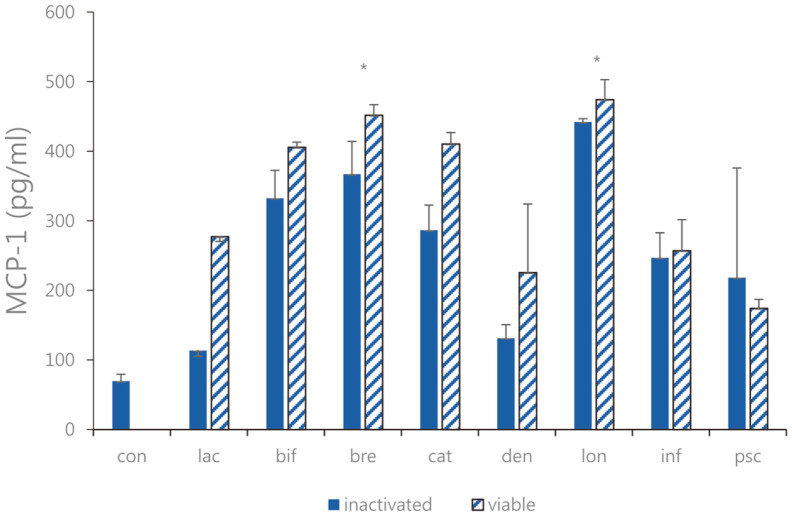
MCP-1 expression of the sonicated *Bifidobacterium* and viable bifidobacteria in Caco-2 cells treated with pathogenic *E. faecalis* ATCC 29212. con: control; lac: *B. lactis*; bif: *B. bifidum*; bre: *B. breve*; cat: *B. catenulatum*; den: *B. dentium*; lon: *B. longum*; inf: *B. infantis*; psc: *B. pseudocatenulatum*. Values are represented as the mean ± S.D. (*p < 0.05).

**Fig. 6:**
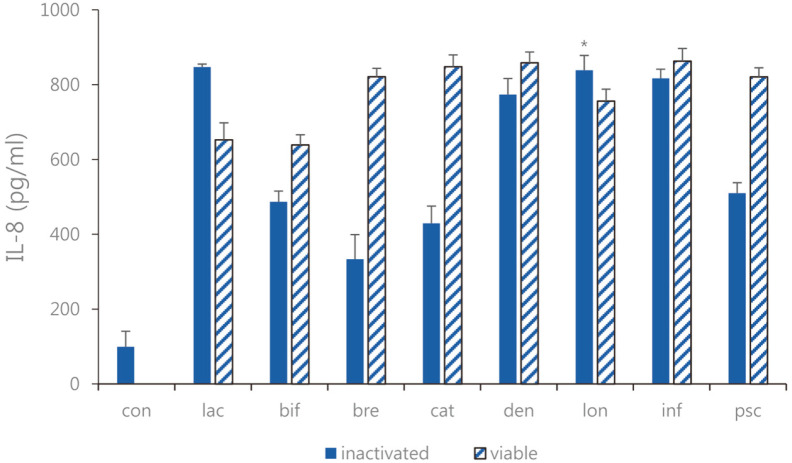
IL-8 expression of the sonicated *Bifidobacterium* and viable bifidobacteria in Caco-2 cells treated with pathogenic *E. faecalis* ATCC 29212. con: control; lac: *B. lactis*; bif: *B. bifidum*; bre: *B. breve*; cat: *B. catenulatum*; den: *B. dentium*; lon: *B. longum*; inf: *B. infantis*; psc: *B. pseudocatenulatum*. Values are represented the mean ± S.D. (*p < 0.05).

Expression of MCP-1, and IL-8 as chemotactic cytokines were measured. We treated LPS-induced Caco-2 cells as described above with 1 × 10^9^ CFU/ml of each strain of sonicated *Bifidobacterium* extracts and viable bifidobacteria, and ELISA was performed. As a result of comparing LPS-induced Caco-2 cells treated with sonicated *Bifidobacterium* extracts and viable bifidobacteria, the expression levels of MCP-1 and IL-8 increased (Figs. 5 and 6). Sonicated *Bifidobacterium* extracts increased IL-8 expression more than viable bifidobacteria. Moreover, both types of *Bifidobacterium* effectively increased the expression of MCP-1. Overall, both viable bifidobacteria and sonicated extracts increased MCP-1 expression, and the regulatory effect of sonicated *Bifidobacterium* extracts was higher than that of viable bifidobacteria. Therefore, among the four lactic acid bacteria, *B. bifidum*, *B. breve*, *B. longum*, and *B. catenulatum*, and *B. breve*, in which the effect of sonicated *Bifidobacterium* extracts was more remarkable, the sonicated sample of *B. breve* showed increasing expression IL-8 by 339.6 ± 36.78 pg/ml compared to the case of the viable bifidobacteria. Furthermore, among the four lactic acid bacteria, *B. bifidum*, *B. breve*, *B. longum*, and *B. catenulatum*, and *B. breve* and *B. longum* in which the effect of sonicated *Bifidobacterium* extracts was more significant, the sonicated sample of *B. breve* and *B. longum* showed increasing expression MCP-1 by 367.1 ± 47.08 pg/ml and 442.1 ± 4.78 pg/ml compared to the case of the viable bifidobacteria sample.

**Fig. 7:**
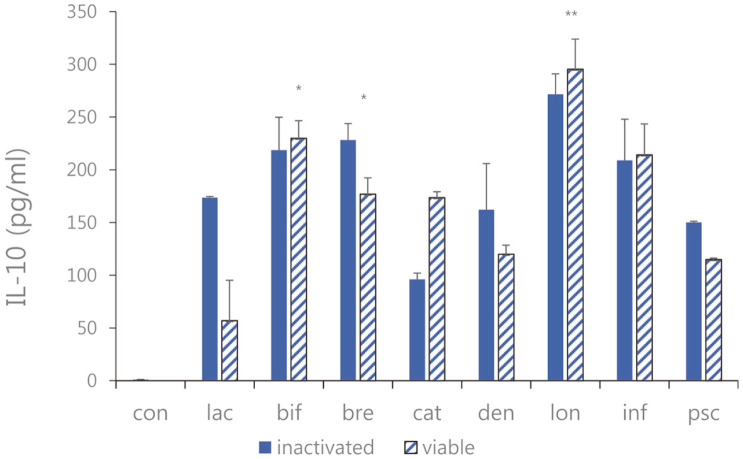
IL-10 expression of the sonicated *Bifidobacterium* and viable bifidobacteria in Caco-2 cells treated with pathogenic *E. faecalis* ATCC 29212. con: control; lac: *B. lactis*; bif: *B. bifidum*; bre: *B. breve*; cat: *B. catenulatum*; den: *B. dentium*; lon: *B. longum*; inf: *B. infantis*; psc: *B. pseudocatenulatum*. Values are represented as the mean ± S.D. (*p < 0.05, **p < 0.01).

Expression of IL-10 as an anti-inflammatory cytokine was measured. As a result of comparing LPS-induced Caco-2 cells treated with sonicated *Bifidobacterium* extracts and viable bifidobacteria, the expression levels of IL-10 were increased (Fig. 7). It was shown that the expression level of IL-10 treated with viable bifidobacteria and the sonicated *Bifidobacterium* extracts were more increased than the positive control. Furthermore, among the four types lactic acid bacteria *B. bifidum*, *B. breve*, *B. infantis* and *B. longum*, in which the effect of sonicated *Bifidobacterium* extracts was more remarkable, the sonicated sample of *B. longum* showed a difference in increasing expression IL-10 by 271.61 ± 19.38 pg/ml compared to the case of the viable bifidobacteria.

### Measurement for Production of NO

**Fig. 8:**
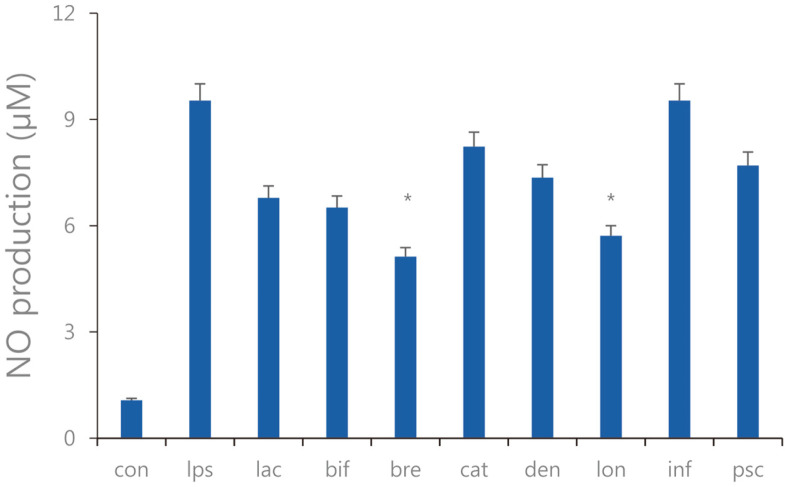
NO production of the sonicated *Bifidobacterium* extracts in the RAW 264.7 cells treated with pathogenic *E. faecalis* ATCC 29212. con: control; lps: lipopolysaccharide, lac: *B. lactis*; bif: *B. bifidum*; bre: *B. breve*; cat: *B. catenulatum*; den: *B. dentium*; lon: *B. longum*; inf: *B. infantis*; psc: *B. pseudocatenulatum*. Values are represented as the mean ± S.D. (*p < 0.05).

We performed an NO assay to confirm whether sonicated cells exhibited anti-inflammatory effects by inhibiting the expression of pro-inflammatory cytokines in RAW 264.7 cells against *E. faecalis* ATCC 29212 infection. Some *Bifidobacterium* spp. showed that NO production decreased or increased regardless of the results of the cytokine expression measurements. Therefore, it is inferred that the reduction in inflammatory cytokine expression does not necessarily substantially affect the mechanism whereby sonicated *Bifidobacterium* extract inhibits NO production. An NO assay was performed to confirm the anti-inflammatory effect of sonicated *Bifidobacterium* extracts. Overall, NO production was decreased in RAW 264.7 cells treated with sonicated *Bifidobacterium* extracts. *B. breve* and *B. longum*, in which the effect of sonicated *Bifidobacterium* extracts was more significantly inhibited NO production by 5.12 ± 0.61 pg/ml and 5.71 ± 0.71 pg/ml compared to LPS-treated negative control group (Fig. 8).

### Evaluation of Clinical Efficacy of *B. breve* and *B. longum*

To evaluate the clinical efficacy of *B. breve* and *B. longum* in acute diarrhea in children, 50 pediatric patients aged 3 months to less than 5 years who were admitted to a university hospital from November 2021 to February 2022 and whom were included in the study after obtaining the informed consent to participate (IRB approval number; 2021-012-017-B). The subjects, who were in the acute diarrhea group, were divided into the *B. breve* (n =17), the *B. longum* (*n* = 19) and the placebo (*n* = 14) group. There was no difference between the three groups in terms of etiology and the *B. breve* and *B. longum* group and the placebo group was matched for age, respectively. The mean duration of vomiting was 2.52 ± 0.45 and 2.33 ± 1.23 days in the *B. breve* and *B. longum* group and 3.16± 1.31 days in the placebo group, but the difference was not significant (*p* = 0.073). The mean duration of diarrhea episodes was 2.29 ± 0.04 and 2.93 ± 0.07 in the *B. breve* and *B. longum* group and 2.56 ± 0.09 in the placebo group, and the difference between the three groups was statistically significant (*p* = 0.068). The mean duration of fever episodes was 2.19 ± 0.49 and 2.72 ± 0.68 in the *B. breve* and *B. longum* groups and 3.57 ± 0.56 in the placebo group, and the difference between the three groups was statistically significant (*p* = 0.289) (Table 2). In the *B. breve* and *B. longum* group, it was observed that diarrhea duration was shortened compared to placebo and did not cause side effects.

## Discussion

Most probiotics are based on the use of cells inactivated by heat treatments, which might modify heat susceptible probiotic proteins. Accordingly, more effective inactivation processes are required, such as sonication [25]. For this reason, it is possible to minimize loss, and maintain efficacy by sonicated treating probiotic LAB as an unheated method. When viable bifidobacteria is an experimental material, it is marked as probiotics, and when sonicated *Bifidobacterium* extract is an experimental material, it is specified as postbiotics. In this investigation, a sonicated *Bifidobacterium* extracts were prepared as postbiotics, and the effect on six immunomodulatory cytokines was measured. Subsequently, efficacy of the *B. breve* and the *B. longum* of sonicated extracts compared with that of viable cells were analyzed on acute diarrhea in children.

Firstly, the expression levels of pro-inflammatory cytokines were measured. The results showed that the expression levels of TNF-α, IL-6, and IL-12, in sonicated *Bifidobacterium* extracts and viable bifidobacteria were reduced. The pro-inflammatory cytokines TNF-α, IL-6, and IL-12 are expressed when LPS is recognized by toll-like receptor-4 (TLR-4), activating the mitogen activated protein kinase (MAPK) family [26]. These cytokines play a critical role in acute inflammatory reactions. Therefore, TNF-α and IL-6 are well known to be related to autoimmune diseases such as rheumatism. IL-12 is a pro-inflammatory cytokine that is involved in the expression of TNF-α and is known to be associated with Crohn's disease. IL-10 is an anti-inflammatory cytokine that inhibits excessive IL-12 production. However, according to the experimental results, increased IL-10 production does not have a direct inhibitor effect on IL-12. *Bifidobacterium* seemed to inhibit the production of IL-12 through a mechanism other than one induced by IL-10. The inhibitory effect against IL-8 was slightly lower compared with that for other cytokines in cells with added LAB, however treatment with sonicated *Bifidobacterium* extracts showed a greater effect than treatment with the viable bacteria. Considering the results, various protein metabolites present inside bifidobacterial cells may have been able to maintain their original shape without being deformed in the process of sonication, thus exhibiting a more significant immunomodulatory effect than viable cells. Nitric oxide (NO) is produced by the oxidation of L-arginine by nitric oxide synthase (NOS) in macrophages stimulated by LPS of Gram-negative bacteria. NO is known to play an important role in the maintenance of cell function and mediate various inflammatory responses such as pain, edema, and fever [27]. NO is produced by neural nNOS (NOS1), endothelial NOS (NOS3), or inducible NOS (NOS2) and is known to be be highly expressed when proinflammatory cytokines such as TNF-α, IL-1, and IL-6 are upregulated. Many studies have reported that probiotics are effective at inhibiting NO generation. In particular, Toumi *et al*. argued that probiotics meaningfully inhibit elevated NO production in backgrounds of Crohn's disease and inflammatory bowel disease [28].

In the present study, we found sonicated *Bifidobacterium* cells and viable cells had similar cytokine controlling effects but that reductions in the expression levels of pro-inflammatory cytokines were not consistent between *Bifidobacterium* strains. Each of the *Bifidobacterium* strains was found to act more specifically for different cytokines. Since some *Bifidobacterium* spp. have not been studied as sonicated-inactivated cells, further studies were required to determine their efficacies and clinical effects. In vitro and in vivo experiments continue to be of increasing interest, however, even if these results are positive, human application experiments may result in different results, although there are relatively few human application test subjects. Orally administered LABs pass through the gastrointestinal tract, activate the intestinal immune system, and increase the production of mucosal IgA, which inhibits intestinal colonization by pathogenic bacteria and the invasion of pathogenic antigens. Furthermore, LAB species have different effects, which suggests that not all LABs are effective and that the usages and dosages of LABs should be tailored according to the cause of diarrhea. We suggest a microbiome library of probiotic intestinal microbial data should be created, that probiotics functions should be subdivided according to individual characteristics, and that personalized probiotics be designed to address individual symptoms. Each individual needs different probiotic strains, and somatic probiotics are customized probiotics that subdivide and develop necessary functions according to individual characteristics and disease characteristics.

In conclusion, as postbiotics, sonicated *Bifidobacterium* extracts showed similar effects to probiotics, and sonicated *Bifidobacterium* postbiotics were found to have effects similar to probiotics, which suggests postbiotics could also be used as pharmaceuticals. but additional clinical trials are required. However, additional clinical trials are required, and more extensive research is needed to determine the usefulness of B. brave as a treatment for diarrhea in children.

## Figures and Tables

**Table 1:** Identification and origin of the probiotic *Bifidobacterium* strains from KCTC and ATCC.

Probiotic strains	Origin		

	ATCC	KCTC	Source
*Bifidobacterium animalis* subsp*. lactis*	25527	5854	Fermented Milk
*Bifidobacterium bifidum*	29521	3202	Infant
*Bifidobacterium breve*	15700	3220	Infant
*Bifidobacterium catenulatum*	27539	3221	Adult
*Bifidobacterium dentium*	27534	3222	Adult
*Bifidobacterium longum*	15707	3128	Adult
*Bifidobacterium infantis*	15697	3249	Infant
*Bifidobacterium pseudocatenulatum*	27919	3223	Infant

**Table 2:** Clinical efficacy and characteristics of the *B. breve* and *B. longum* on acute diarrhea in children.

	*B. breve* (*n* = 17)	*B. longum* (*n* = 19)	Placebo (*n* = 14)	*P* value
Vomiting				
Frequency (%)	8.4 (53.7%)	10.6 (55.6%)	7 (51%)	0.31
Duration (Day)	2.5	2.3	3.1	0.07
Diarrhea				
Frequency (%)	21 (30.7%)	21 (33.7%)	5 (43.7%)	0.04
Duration (Day)	2.2	2.9	2.5	0.06
Fever				
Frequency (%)	7.6 (43.7%)	9.8 (45.7%)	4 (59.7%)	0.72
Duration (Day)	2.1	2.7	3.5	0.29
Sex				0.53
Male	10	11	8	
Female	7	8	6	
Age (Month)	27.5.6 ±17.36	25.4 ± 28.27	29.7± 24.28	0.63
